# The Effect of Topophysis on the In Vitro Development of *Handroanthus guayacan* and on Its Metabolism of Meta-Topolin Riboside

**DOI:** 10.3390/plants12203577

**Published:** 2023-10-15

**Authors:** Maroua Grira, Els Prinsen, Stefaan P. O. Werbrouck

**Affiliations:** 1 Laboratory for Applied In Vitro Plant Biotechnology, Ghent University, Valentin Vaerwyckweg 1, 9000 Ghent, Belgium; 2 Integrated Molecular Plant Physiology Research, Dement of Biology, University of Antwerp, Groenenborgerlaan 170, 2020 Antwerp, Belgium

**Keywords:** cytokinin metabolites, N-glucosides, O-glucosides, *Handroanthus guayacan*, topophysis, micropropagation medium

## Abstract

An important factor affecting the uniformity of in vitro cultures is the topophysical position of the original explant. We investigated this phenomenon in *Handroanthus guayacan*, a tropical woody tree species. Shoots from a stock culture were separated into upper, middle and basal sections and transferred to a modified MS medium containing meta-topolin-riboside and indole-butyric acid. After 8 weeks, the middle section produced the most shoots, the longest shoots and the highest number of nodes per plant. Shoots derived from the upper section were elongated, but had the shortest internodes, while those from the basal section formed the largest callus. None of the three types of explants rooted during the proliferation phase. The topophysically dependent spatial distribution of endogenous cytokinins and auxins was determined. The topophysical effect observed could not be explained solely by analyzing the endogenous isoprenoid and auxin. However, the metabolism and distribution of the aromatic cytokinin could provide an explanation. The concentration of the meta hydroxy-substituted topolins was highest in shoots derived from the middle section. Aromatic N- and O-glucosides were much more concentrated in the leaves than in the stems. In conclusion, it is recommended to consider the explant’s topophysis when developing a multiplication protocol to avoid heterogeneity in an in vitro culture.

## 1. Introduction

Due to the uncontrolled overexploitation of tropical timber species in recent decades, numerous tree species are threatened with extinction. The scarcity of seeds and their poor germination rates often cause delays in reforestation programs. In vitro propagation is the only alternative by which to produce sufficient plant material. When developing a protocol specific to a particular species, emphasis is often placed on the composition of the base medium and the concentration of cytokinins (CKs), while little consideration is given to physiological variations within the in vitro plant. Neglecting these variations can lead to heterogeneity in the growth of the in vitro culture. Topophysis refers to the influence of shoot position on the growth and development of a cutting [1]. This study aimed to investigate the influence of topophysis position on the in vitro plant growth of an explant and the possible correlation with auxins and CKs along the shoot, which has rarely been investigated. In addition, we wanted to shed light on the metabolism of meta-topolin-riboside (mTR), a cytokinin not commonly used in the micropropagation of woody plants. In vitro, CKs play a role in initiating shoot meristems, promoting proliferation, inhibiting apical dominance and delaying senescence. They also prevent rooting and induce callus formation [2,3]. CKs include natural and synthetic purine derivatives: isoprenoid CKs (ISCKs such as trans-zeatin (tZ), cis-zeatin (cZ), N^6^-isopentenyl adenine (iP), and dihydrozeatin (DZ)) and aromatic CKs (ARCKs, such as N^6^-benzyladenine (BA), kinetin (Kin), meta-topolin (mT), para-topolin (pT) and ortho-topolin (oT)) [4,5]. Free bases are active [6], but once they are ribosylated, they are converted to transport forms with a weaker cytokinin activity. O-glucosylation deactivates CKs, but these can be reactivated again after β-glucosidase activity to the corresponding aglycons, free bases and ribosides [7]. N-glucosylation, however, irreversibly deactivates CKs because these glycosides are not β-glucosidase substrates [8,9]. In recent decades, BA has been the most widely used cytokinin in the in vitro industry due to its availability and affordability [10]. However, its high concentrations or prolonged use can lead to somaclonal variations [11,12], hyperhydricity [13], root inhibition and callus formation [14]. Topolins, such as mTR, are considered a reasonable alternative, especially in the case of woody plants [15]. The experiments presented were tested on *Handroanthus guayacan*, a tropical woody tree previously called *Tabebuia guayacan* [16,17,18], belonging to the family of Bignoniaceae and is listed in the IUCN’s Red List [19]. In the tropics, the plant is valued for its extensive yellow blooms in response to rainfall. Worldwide, this species is valued for its olive to reddish brown wood that is internationally traded. The interest in this species dates back to pre-Columbian time (AD 880 to 1020), when its wood was utilized as grave gifts by the upper class [18]. *Handroanthus* wood has been found to be resistant to tropical, temperate soil pathogens [20], and marine borer insects [21]. Because the wood is so hard and heavy, it has a great commercial value [22]. *H. guayacan* wood, also known as “Ipe” wood, is highly valued for its density, which exceeds that of concrete and steel, as well as its fire resistance [23].

## 2. Results

### 2.1. Morphological Growth Parameters

**Figure 1:**
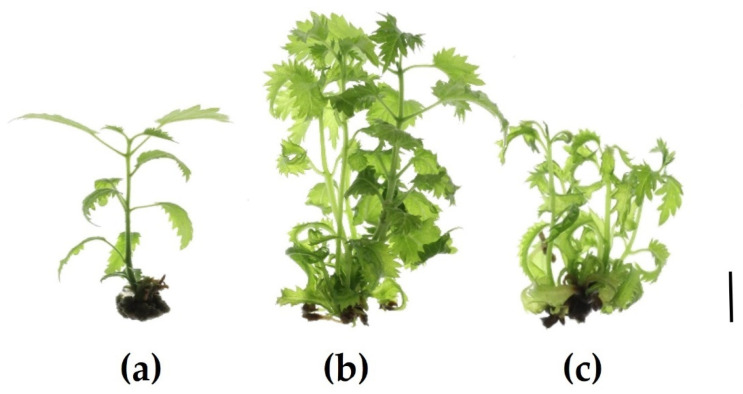
*H. guayacan* in vitro plant developed from an explant of (**a**) upper section; (**b**) middle section; (**c**) basal section (Scale bar is 1 cm).

Morphological growth parameters are shown in Table 1. Shoots derived from the upper section (SUSs) did not branch, but only elongated (Figure 1a). The highest number of shoots (3.8) was obtained from shoots derived from the middle section (SMSs) (Figure 1b). These were also the longest (3.97 cm) and had the highest number of nodes per plant (18.86) compared with 10.15 and 5.35 for shoots derived from the basal section (SBSs) (Figure 1c) and the SUSs (Figure 1a), respectively. The SUSs had the shortest internode length, 0.51 cm, compared with 0.84 cm for the SMSs, which incidentally formed the lightest callus (0.36 g). Leaves at the middle and lower internodes showed leaf epinastic curvature. This effect was more pronounced in SMSs and SBSs.

### 2.2. Hormone Analysis

Auxin and ARCKs were determined. Reported results were limited to values above the detection limit. For convenience, they were divided in frame of activity into relevant groups and summed. ARCKs included aromatic free bases and their ribosides isomers, topolin-O-glucosides, aromatic N-glucosides and para-topolin-riboside (pTR).

#### 2.2.1. Shoots Derived from the Upper Section (SUSs)

**Figure 2:**
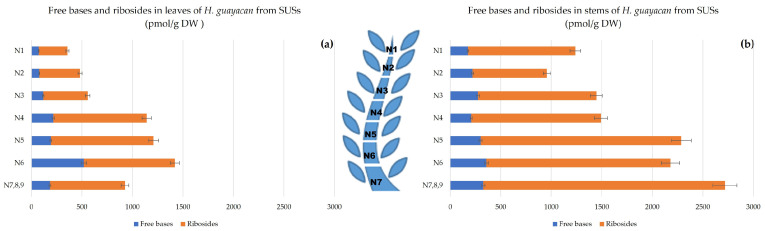
Total amount of aromatic free bases (BA, mT, oT, MemT and MeoT) and their corresponding ribosides (BAR, mTR, oTR, MemTR and MeoTR) in *H. guayacan* derived from SUSs (Shoots derived from the upper section) expressed in pmol/g DW in (**a**) leaves; (**b**) stems.

- Aromatic Cytokinin Free Bases and Riboside Isomers;

The sum of aromatic free bases (N^6^-benzyladenine (BA), meta-topolin (mT), ortho-topolin (oT), meta-methoxy-topolin (MemT), and ortho-methoxy-topolin (MeoT)) followed an ascending gradient from the top (N1) to the base (N7,8,9) as shown in Figure 2a,b. Their concentration ranged from 72 to 519 pmol/g DW in leaves and from 175 to 357 pmol/g DW in the stem and thus were at similar levels. Additionally, the sum of their corresponding ribosides (N^6^-benzyladenosine (BAR), meta-topolin-riboside (mTR), ortho-topolin-riboside (oTR), meta-methoxy-topolin-riboside (MemTR), and ortho-methoxy-topolin-riboside (MeoTR) followed the same trend and increased from top to bottom, with higher concentrations in the stem than in the leaves (between 1100–2400 pmol/g DW and 300–1000 pmol/g DW, respectively).

**Figure 3:**
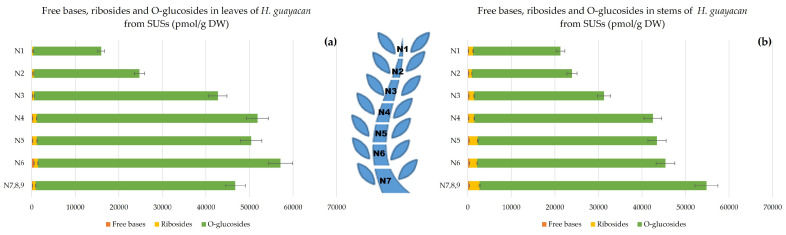
Total amount of aromatic free bases (BA, mT, oT, MemT and MeoT), ribosides (BAR, mTR, oTR, MemTR and MeoTR) and O-glucosides (mTROG, pTROG, mTOG and pTOG) in *H. guayacan* derived from SUSs (shoots derived from the upper section) expressed in pmol/g DW in (**a**) leaves and (**b**) stems.

- Topolin-O-Glucosides;

Topolin-O-glucosides contain the following inactive, but reversible, cytokinin storage forms: meta-topolin-riboside-O-glucoside (mTROG), ortho-topolin-riboside-O-glucoside (oTROG), para-topolin-riboside-O-glucoside (pTROG), meta-topolin-O-glucoside (mTOG), ortho-topolin-O-glucoside (oTOG), and para-topolin-O-glucoside (pTOG). All the mentioned aromatic O-glucosides, except oTROG and oTOG, were detected in *H. guayacan* extracts. Figure 3 showed that topolin-O-glucosides are present in large amounts, with no obvious differences between the stem (Figure 3b) and the leaves (Figure 3a). In the leaves, the O-glucosides are 48–96 times more concentrated than the riboside aglycon and 107–356 times more concentrated than the free base aglycons. In the stem, their concentration was found to be 19–32 times higher than ribosides and 104–197 times higher than the free bases. Topolin-O-glucosides were found to be higher at the base of the shoot than at the top, and their gradient was the same in both leaves and stem, reaching a maximum of 55,707 pmol/g DW in leaves and a maximum of 52,093 pmol/g DW in stem.

**Figure 4:**
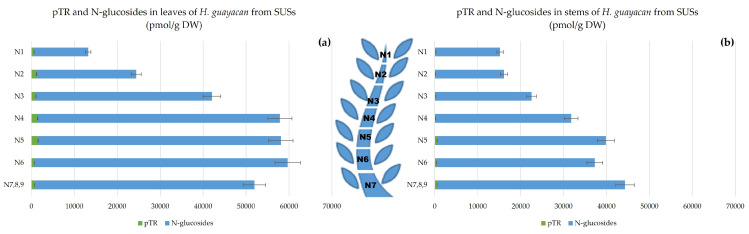
Total amount of aromatic N-glucosides (BA3G, BA7G, mT9G and oT9G) and pTR in *H. guayacan* derived from SUSs (shoots derived from the upper section) expressed in pmol/g DW in (**a**) leaves and (**b**) stems.

- Aromatic Cytokinin N-Glucosides and pTR;

N-glucosides are considered to be inactive irreversible forms, and include N^6^-benzyladenine-3-glucoside (BA3G), N^6^-benzyladenine-7-glucoside (BA7G), N^6^-benzyladenine-9-glucoside (BA9G), meta-topolin-7-glucoside (mT7G), meta-topolin-9-glucoside (mT9G), ortho-topolin-7-glucoside (oT7G), ortho-topolin-9-glucoside (oT9G), para-topolin-7-glucoside (pT7G) and para-topolin-9-glucoside (pT9G). Their synthesis is considered a detoxification process of the plant. All of them were detected, except for BA9G, oT7G, mT7G, pT7G and pT9G. Figure 4 represents the total concentration of N-glucosides together with pTR (also considered inactive). These occurred in huge concentrations in the leaves, ranging from 12,600 pmol/g DW in the highest leaves to 59,000 pmol/g DW in the lowest leaves (Figure 4a). The concentration was similarly high in the stem (Figure 4b), ranging from 15,000 pmol/g DW in the top of the stem (N1) to 44,000 pmol/g DW at the base (N7,8,9). The inactive pT was below the detection limit but its riboside pTR was well quantified and displayed a concentration between 660 and 1600 pmol/g DW in leaves (N1–N5) and slightly lower in the stem, between 200 and 570 pmol/g DW (N2–N5).

- Auxins.

**Figure 5:**
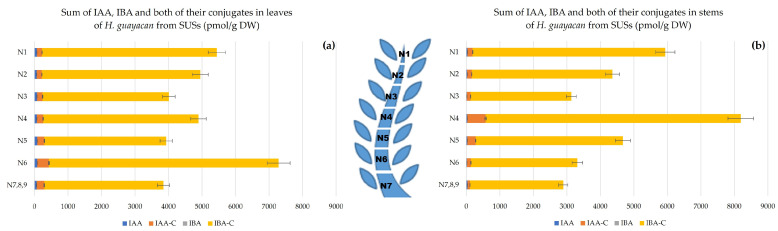
Auxins (sum of IAA, IBA and the total amount of both their conjugates) in *H. guayacan* derived from SUSs (plant derived from the upper section) expressed in pmol/g DW in (**a**) leaves and (**b**) stems.

Auxins, including indole-3-acetic acid (IAA), indole-3-butyric acid (IBA) and the total amount of their conjugates, respectively IAA- and IBA-conjugates, were quantified. The auxin concentration was highest at the apex, with a decreasing gradient toward the base (Figure 5a), and with similar concentrations in leaves and stems (Figure 5b). In leaves, the highest concentration occurred in the upper leaves (about 5500 pmol/g DW), and 3800 pmol/g DW in the leaves of the lower node (N7,8,9). In the stems, the total amount of auxins ranged from nearly 6000 pmol/g DW in N1 to nearly 3000 pmol/g DW in N7,8,9.

#### 2.2.2. Shoots Derived from the Middle Section (SMSs)

**Figure 6:**
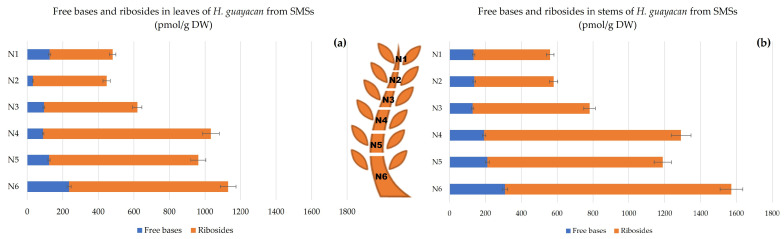
Total amount of aromatic free bases (BA, mT, oT, MemT and MeoT) and their corresponding ribosides (BAR, mTR, oTR, MemTR and MeoTR) in *H. guayacan* derived from SMSs (shoots derived from the middle section) expressed in pmol/g DW in (**a**) leaves and (**b**) stems.

- Aromatic Cytokinin Free Bases and Riboside Isomers;

In shoots derived from the middle section (SMSs), the active free bases were more concentrated in the stem (Figure 6b) than in the leaves (Figure 6a): 32–240 pmol/g DW (in N2 and N6, respectively) compared with 128–300 pmol/g DW for the stem (in N3 and N6). Depending on the position of the node along the shoot, the amounts of ribosides in leaves and stem were 3 to 13 times higher and 3 to 6 times higher, respectively, compared with the corresponding free bases. For both aromatic active forms (free bases and ribosides), the gradient in both leaves and stem was the same.

**Figure 7:**
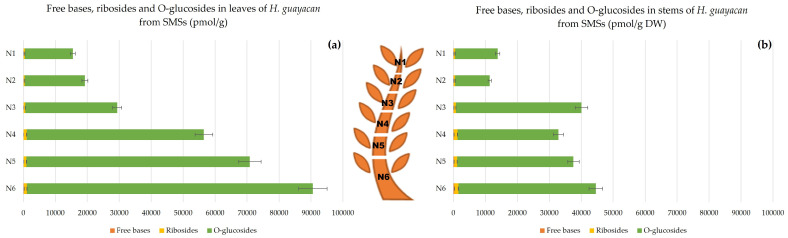
Total amount of aromatic free bases (BA, mT, oT, MemT and MeoT), ribosides (BAR, mTR, oTR, MemTR and MeoTR) and O-glucosides (mTROG, pTROG, mTOG and pTOG) in *H. guayacan* derived from SMSs (shoots derived from the middle section) expressed in pmol/g DW in (**a**) leaves and (**b**) stems.

- Topolin-O-Glucosides;

In SMSs, topolin-O-glucosides showed an increasing concentration from the top of the shoot to the bottom, where they reached an impressive concentration at the base of the plant: in leaves the highest concentration reached nearly 90,000 pmol/g DW (in N6) (Figure 7a), and 43,000 pmol/g DW in stem (N6) (Figure 7b). The ratio of O-glucosides to ribosides varied between a minimum of 42 and a maximum of 100 for leaves and between 24 and 60 for stem. Moreover, the ratio of O-glucosides to free bases varied between 120 and 622 for leaves and between 79 and 306 for stem.

**Figure 8:**
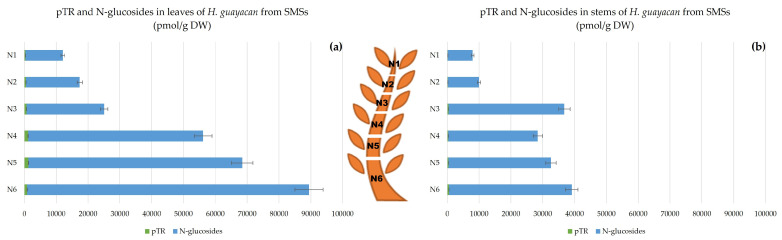
Total amount of aromatic N-glucosides (BA3G, BA7G, mT9G and oT9G) and pTR in *H. guayacan* derived from SMSs (shoots derived from the middle section) expressed in pmol/g DW in (**a**) leaves and (**b**) stems.

- Aromatic N-Glucosides and pTR;

pT was below the detection limit, but the ribosylated form pTR was detectable, its concentrations being almost negligible compared with N-glucoside concentrations (Figure 8). For SMSs, leaves contained remarkably high levels of N-glucosides, ranging from 11,000 in N1 to nearly 89,000 pmol/g DW in N6 (Figure 8a). In the stem, concentrations did not exceed half of those in leaves (nearly 38,600 pmol/g DW). In stems, the ratio of N-glucosides to pTR was 60–98 times higher (Figure 8b). Both aromatic N-glucosides and pTR accumulated more at the base of SMSs.

- Auxins.

**Figure 9:**
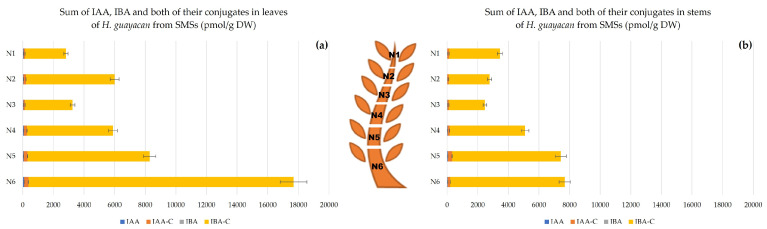
Auxins (sum of IAA, IBA and the total amount of both their conjugates) in *H. guayacan* derived from SMSs (plant derived from the middle section position) expressed in pmol/g DW in (**a**) leaves and (**b**) stems.

In contrast with SUSs, the concentration of auxins in SMSs showed a clear acropetal gradient. In leaves (Figure 9a), the highest summed concentration (nearly 18,000 pmol/g DW) was produced in the lower leaves (N6). The lowest concentration, nearly 3000 pmol/g DW, was produced in the upper leaves of N1. In the stem (Figure 9b), the auxin concentration followed the same decreasing gradient from the top to the base of the shoot. At the apex (stem of N1) 3452 pmol/g DW of auxin and its conjugates were measured, and at the stem of N6 up to 7698 pmol/g DW (Figure 9b).

#### 2.2.3. Shoots Originating from the Basal Section (SBSs)

**Figure 10:**
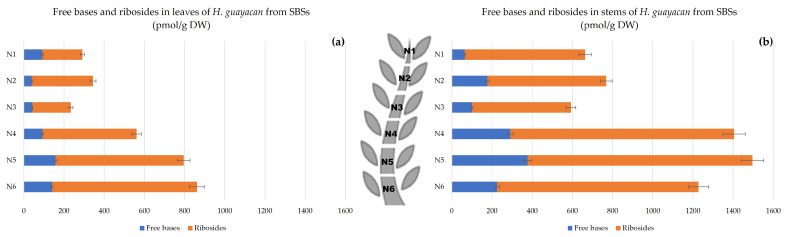
Total amount of aromatic free bases (BA, mT, oT, MemT and MeoT) and their corresponding ribosides (BAR, mTR, oTR, MemTR and MeoTR) in *H. guayacan* derived from SBSs (shoots derived from the basal section) expressed in pmol/g DW in (**a**) leaves and (**b**) stems.

- Aromatic Cytokinin Free Bases and Riboside Isomers;

The SBSs also showed a decreasing acropetal gradient in both leaves (Figure 10a) and stems (Figure 10b). The concentrations of aromatic free bases varied between 40 and 160 pmol/g DW and between 60 and 380 pmol/g DW in the leaves and stem, respectively. The concentrations of their corresponding ribosides were much higher, with a ratio to free bases that was up to eight times higher in the leaves and nine times higher in the stem.

**Figure 11:**
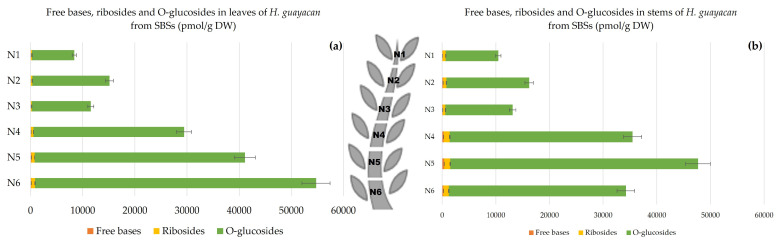
Total amount of aromatic free bases (BA, mT, oT, MemT and MeoT), ribosides (BAR, mTR, oTR, MemTR and MeoTR) and O-glucosides (mTROG, pTROG, mTOG and pTOG) in *H. guayacan* derived from SBSs (shoots derived from the basal section) expressed in pmol/g DW in (**a**) leaves and (**b**) stems.

- Topolin-O-Glucosides;

The concentration of topolin-O-glucosides increased from 8100 pmol/g DW in the upper leaves of N1 to nearly 54,000 pmol/g DW in the lower leaves of N6 (Figure 11a). In the stem, it varied from a minimum 9794 pmol/g DW in N1 to a maximum of 46,200 pmol/g DW in the stem of N5 (Figure 11b).

**Figure 12:**
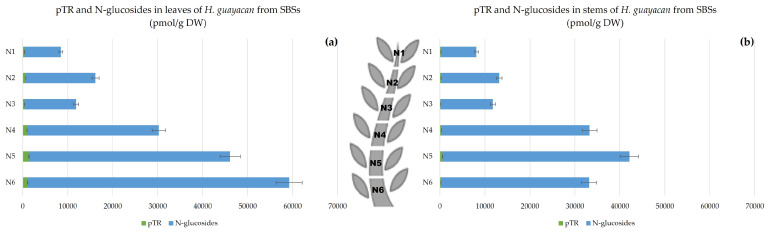
Total amount of aromatic *N*-glucosides (BA3G, BA7G, mT9G and oT9G) and pTR in *H. guayacan* derived from SBSs (shoots derived from the basal section) expressed in pmol/g DW in (**a**) leaves and (**b**) stems.

- Aromatic Cytokinin N-Glucosides and pTR;

In SBSs, *N*-glucosides were measured in both leaves and stem. Figure 12 shows a clear decreasing acropetal gradient. In leaves, the concentration ranged from 8070 pmol/g DW in N1 to 58,122 pmol/g DW in N6 (Figure 12a). However, the concentrations in the stem were lower, ranging from a minimum of 7911 to a maximum of 41,531 pmol/g DW in N1 and N6, respectively (Figure 12b).

- Auxins.

**Figure 13:**
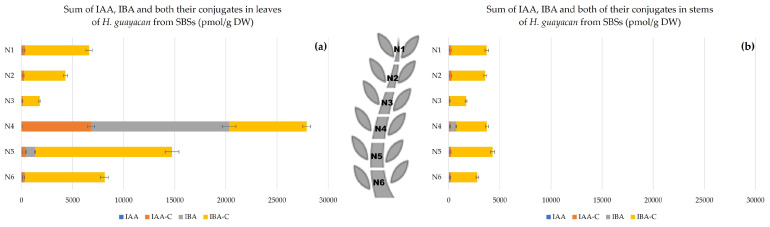
Auxins (sum of IAA, IBA and the total amount of both their conjugates) in *H. guayacan* derived from SBSs (plant derived from the basal section) expressed in pmol/g DW in (**a**) leaves and (**b**) stems.

There was no clear gradient present in leaves and stem (Figure 13). In stem, there was almost no difference in concentration along the plant: 3726 and 2806 pmol/g DW in stem of N1 and N6, respectively. In leaves, a notable peak was observed in the middle leaves of N4.

### 2.3. Meta, Ortho and Para Isomers of CK Metabolites

**Figure 14:**
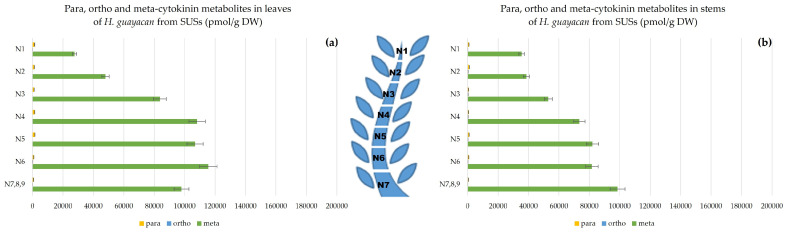
Meta, ortho and para hydroxy-substituted cytokinin metabolites in *H. guayacan* derived from SUSs expressed in pmol/g DW in (**a**) leaves and (**b**) stems.

**Figure 15:**
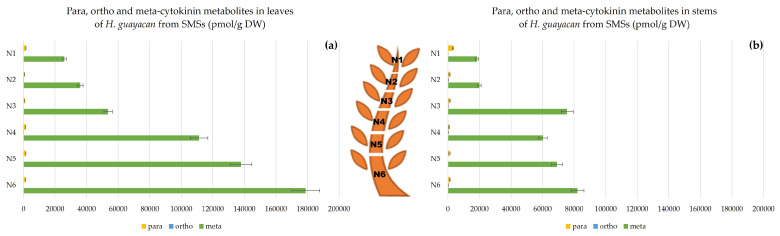
Meta, ortho and para hydroxy-substituted cytokinin metabolites in *H. guayacan* derived from SMSs expressed in pmol/g DW in (**a**) leaves and (**b**) stems.

**Figure 16:**
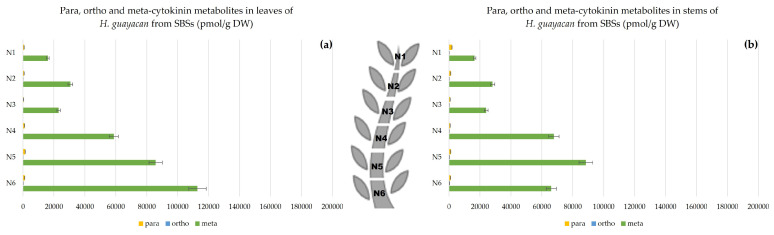
Meta, ortho and para hydroxy-substituted cytokinin metabolites in *H. guayacan* derived from SBSs expressed in pmol/g DW in (**a**) leaves and (**b**) stems.

Figure 14, Figure 15 and Figure 16 show the difference between meta, ortho and para hydroxy-substituted compounds in leaves and stem of the three topophysical positions. For SMSs, the meta position isomers were most concentrated in leaves: nearly 180,000 pmol/g DW (Figure 15a), compared with their concentration in leaves of both SUSs (Figure 14a) and SBSs (Figure 15a) with a maximum of 115,440 and 112,713 pmol/g DW, respectively. In the stem, the meta position at the base of the shoot reached a maximum of 82,174 pmol/g DW for SMSs (Figure 15b), 98,540 pmol/g DW for SUSs (Figure 14b) and 88,464 pmol/g DW for SBSs (Figure 16b).

## 3. Discussion

Topophysis has been studied mainly in relation to the rooting capacity of cuttings [24,25,26]. Only a small number of reports are available on the effects of topophysis in in vitro applications [27,28,29] whereas no studies are available on the relationship between topophysis and cytokinin metabolism of plant material grown in vitro. This study shows that shoots derived from nodes located in the middle section of the shoot were the most productive and had the highest number of shoots, the greatest length of shoots and the highest number of nodes per plant. Separating the explants based on their topophysical position may solve the problem of heterogeneity in the in vitro culture of *Handroanthus guyacan*.

Endogenous ISCKs were present in low concentrations and did not explain the topophysis effect (results are not shown). In general, cis-zeatin 9-riboside (cZR) and N^6^-(Δ2-isopentenyl) adenine 9-glucoside (iP9G) occurred in leaves and stems. N^6^-isopentenyladenine riboside (iPR) was present only in the stem, in contrast with zeatin-O-glucoside (ZOG), which was mainly detected in the leaves. ARCKs were found to have a greater influence than IRCKs on developmental processes such as morphogenesis [30,31]. This is consistent with Hosek et al. [7] for Arabidopsis, and Uzelac et al. [32] for tobacco. This finding is not unexpected as previous experiments have shown that mTR gives the best results for in vitro multiplication and that mTR is indeed used as an exogenous cytokinin to generate the material. Despite the fact that the material was grown on exogenous mTR, free bases and that ribosides were almost negligible (<0.5%), the most abundant metabolites in leaves and stems were N- and O-glucosides (each accounting for about 50%). The concentration of mTR in the culture medium was 5 µM, whereas the concentration in the middle and basal explants of the O- and N-glucosides easily reached concentrations 10 times higher, indicating the bioaccumulative nature of these glucosides. The possible slow breakdown of O-glucosides into free bases may explain their role in inducing the significant shoot proliferation observed. Especially in the leaves of SMSs, N- and O-glucosides reached concentrations of 89 and 90 µM, respectively, whereas half this level was found in SUSs and SBSs. In general, all cytokinin metabolites showed an increasing gradient toward the basal region of shoots. We assume that this was also true for the original shoots, which were divided into three sections to obtain the explants. The explants received fresh mTR from the culture medium and accumulated significant amounts of mainly O- and N-glucosides. Although CK N-glucosides do not activate CK receptors, Hoyerova and Hosek [33] have suggested that the N7- and N9-glucosides of trans-zeatin (tZ) can be metabolized to release free CK bases. In addition, they have shown that CK N-glucosides are likely to play a role as transportable CK metabolites. The idea that aromatic CK-N-glucosides may play a similar role is also not excluded.

According to Dolezal et al. [34], the meta hydroxy-substituted isomers were more active than the ortho isomers while the para position was the least active. Considering that the explants are grown on mTR, it is not surprising that the meta hydroxy-substituted isomers are the most abundant, but the plant can change the hydroxy position to the ortho or para position. The presence of oT and pT has also been reported by Aremu et al. [31] in micropropagated bananas and Abdouli et al. [35] in in vitro pistachio. This mechanism can be considered a detoxification strategy.

Auxin is synthesized in the shoot apex and young leaves. This study confirms a slight polar basipetal gradient of auxin in both leaves and stems of shoots derived from SUSs. However, the gradient was reversed in the leaves and stem of SMSs and disordered in the shoots of SBSs in both leaves and stems. It is important to note that the observed auxin concentrations are the net result of both endogenous auxin (IAA and IBA) biosynthesis and uptake, with consecutive conjugation and transport of the auxins derived from the exogenous IBA taken up from the medium. Taking into account this additional exogenous auxin source, the total auxin pool will participate in the regulation of auxin steady state and will influence the endogenous biosynthesis through feed-back mechanisms. Plant material derived from basal section and middle section will contain relatively more auxins originating from the exogenous source than material derived from SUSs. In this context, the basipetal auxin gradient in SUSs might be the consequence of relatively more endogenous auxins derived from endogenous biosynthesis in the apex (apical source), whereas auxins in SBSs and SMSs that showed a reversed acropetal gradient reflected endogenous auxins from the major basal exogenous source. It is clear from the data reported in this study that the amount of free auxins (IAA as well as IBA) remains constant and low, whereas elevated levels are entirely due to enhanced conjugated metabolites. The leaf epinasty curvature observed in the lower and middle leaves in the shoots derived from SMSs and SBSs explants, may be correlated with the high concentration of auxin (IBA) conjugates visible in the auxin gradient along the consecutive internodes. This is in accordance with Spena et al. [36] who showed that auxin conjugation triggered leaf epinastic curvature, even when the free auxin concentrations is not altered. Thus, it appears that the absolute concentration of auxin is an important trigger for leaf epinastic curvature. Reemmer and Murphy [37] have shown that the auxin gradient was not only a transport gradient from zone A to zone B, but also a critical factor in tissue development. Our results do not explain the lack of shoot production of the SUSs, only based on the endogenous auxin concentrations. Both the exogenous auxins and aromatic cytokinins available as transportable conjugates reach the top zone less easily, resulting in lower total concentrations and, as a consequence, less callus and shoot formation and less pronounced leaf epinastic curvature. It is worth remembering, especially in the context of tissue development, that it is not the absolute concentrations of auxins or cytokinins that matter, but rather it is the ratio of the two that drives differentiation. Due to the dominant presence of the aromatic O-glucosides and O-ribosides in shoots grown from SBSs and SMSs explants, the ratio is in favor of cytokinins.

## 4. Materials and Methods

**Figure 17:**
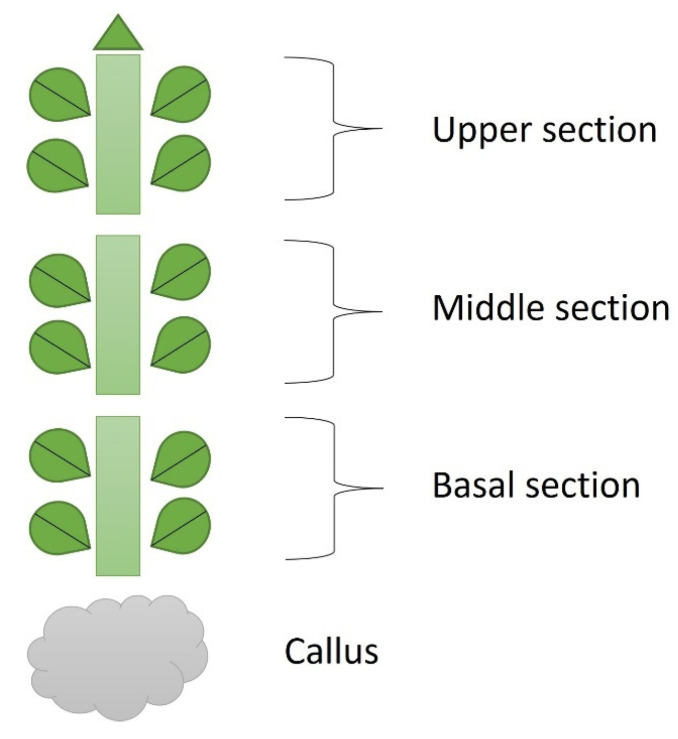
Topophysical zones on the stock plant used for the experiments.

**Figure 18:**
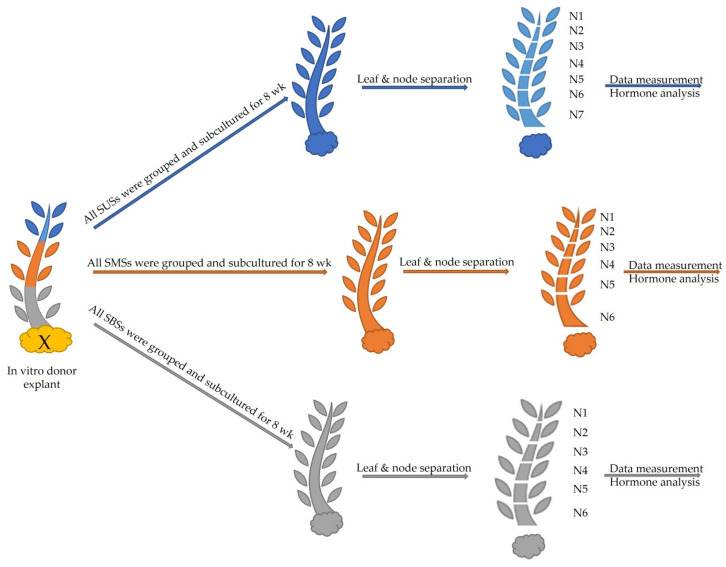
Experimental setup and sample identification of the 3 topophysical positions: upper section in blue color, middle section in orange and basal section in gray.

Explants were taken from three different topophysical zones of in vitro donor shoots of *Handroanthus guyacan*: the upper, middle and basal shoot section (close to, but not including the callus) (Figure 17). Mother plants were provided by Greenlab Biotechnology, Panama. Each explant had two nodes (1.5 cm) and was transferred to a modified Murashige and Skoog medium (Murashige and Skoog, 1962) with half concentration of NH_4_NO_3_ and KNO_3_. This basal salt and vitamin solution was supplemented with 30 g L^−1^ sucrose, 7 g L^−1^ plant agar (Duchefa Biochemie, Haarlem, The Netherlands), 50 mg L^−1^ myo-inositol (Duchefa Biochemie, Haarlem, The Netherlands), 0.25 mg L^−1^ thiamine HCl (Thermo Fisher Scientific, Waltham, MA, USA), 0.5 mg L^−1^ PABA (Merck Sigma-Aldrich, Hoeilaart, Belgium), 1 mg L^−1^ ascorbic acid (Duchefa Biochemie, Haarlem, The Netherlands), 0.01 mg L^−1^ IBA (Duchefa Biochemie, Haarlem, The Netherlands) and 1.85 mg L^−1^ mTR (Olchemim, Olomouc, Czech Republic). The pH was adjusted to 5.8 before autoclaving. The containers used for this study were 720 mL glass vessels with screw-polycarbonate lids. They were maintained in a growth chamber at 26 ± 2 °C under a 16 h photoperiod. Cool fluorescent light was provided by PHILIPS master TLD 36 W 830 Reflex ECO (40 μmol m^−2^ S^−1^ PAR). After 8 weeks of subculture, the following parameters were recorded: Shoot Number (SN), Shoot Length (SL), Node Number (NN) per shoot and per plant, Callus Weight (CW), and Internode Length (IL). To understand the physiological effect of these 3 positions on the growth of the in vitro culture, nodes and leaves were analyzed for endogenous ARCKs and ISCKs. After being grown in vitro for 8 weeks, leaves and stem were separated from each node from the top of the plant to the bottom, excluding callus (Figure 18). Per node, leaves and stem were measured separately. This separation was applied to all nodes of all shoots of the 3 topophysical positions. N1 is the upper node of each shoot in all treatments.

For hormone analysis: 25–300 mg of lyophilized plant material was homogenized and mixed with 1 mL of extraction solvent (80% (*v*/*v*) methanol (HiPerSolv CHROMANORM^®^; VWR, Leuven, Belgium), 20% water (HiPerSolv CHROMANORM^®^; VWR, Leuven, Belgium), followed by a 15 min sonication and overnight extraction at −20 °C. The following internal standards were added for recovery purposes: 300 pmol 3-[^13^C_6_]indolyl-acetic acid ([^13^C_6_]-IAA) (Cambridge Isotope Laboratories, Andover, MA, USA), 70 pmol [^2^H_3_]dihydro-zeatin, [^2^H_3_]dihydro-zeatin riboside, [^2^H_5_]trans-zeatin-7-Glucoside, [^2^H_5_]trans-zeatin-9-glucoside, [^2^H_6_]N6-isopentenyladenine, [^2^H_6_]trans-zeatin-O-glucoside, [^2^H_6_]trans-zeatin-O-glucoside Riboside, [^2^H_6_]N6-isopentenyladenosine, [^2^H_6_]N6-isopentenyladenine-7-glucoside, ^[2^H_6_]N6-isopentenyladenine-9-Glucoside (Olchemim, Olomouc, Czech Republic), ([^2^H_7_]N6-benzyladenine (D-BA), [^2^H_7_]N6-benzyladenosine (D-BAR), [^2^H_7_]N6-benzyladenine-9-glucoside (D-BA9G), ([^15^H_4_]meta-Topolin (^15^N-mT), [^15^N_4_] ortho-Topolin (^15^N-oT), Olchemim, Olomouc, Czech Republic). Oasis^®^ HLB Sorbent (10 mg, Waters, Antwerp, Belgium) was added after overnight extraction to remove pigments. After centrifugation (Eppendorf 5810R (14,000 RPM, 4 °C, 15 min), VWR, Leuven, Belgium), we split the extract into four different aliquots: fraction A (CK), fraction B (IAA, IBA) and fraction C (total-IAA). Fraction A was filtered through a Chromafil^®^ AO-20/3 filter (nylon, pore size 0.20 μm, diameter 3 mm; Macherey-Nagel, Dueren, Germany). The filtrate was collected in an LC-MS vial and kept at 4 °C. ISCKs and ARCKs were analyzed in two separate UPLC-MS/MS runs using separate compound specific solvent gradients. Isoprenoid cytokinins and aromatic cytokinins were analyzed during separate injections of fraction A.

ISCKs were analyzed in fraction A by ACQUITY UPLC coupled ES(+) TQD Tandem Quadrupole UPLC/MS/MS (Waters) in multiple reactant monitoring (MRM) mode using a BEH C18 Column (130Å, 1.7 µm, 2.1 mm × 50 mm) coupled to a BEH C18 VanGuard Pre-column (130Å, 1.7 µm, 2.1 mm × 5 mm), using the following linear gradient: 95:5 A:B with A 0.1 M ammonium acetate (molecular biology grade 7.5 M, Merck Sigma-Aldrich, Hoeilaart, Belgium) in water (HiPerSolv CHROMANORM^®^; VWR, Leuven, Belgium) and B methanol (MeOH) (HiPerSolv CHROMANORM^®^; VWR, Leuven, Belgium), isocratic 95:5 A:B for 0,4 min, 95:5 A:B to 76:24 A:B in 3 min, isocratic 76:24 A:B for 2 min, flow 0.39 µL min^−1^, column temperature 50 °C, injection volume 6 µL. All cytokinins were characterized based on comparisons of the fragmentation characteristics and retention times with the corresponding pure standards (Olchemim, Olomouc, Czech Republic).

ARCKs were analyzed in a second separate UPLC-TQD MS/MS run of fraction A, using the same guard and analytical column as described above for isoprenoid cytokinins. The following chromatographic conditions were used: 95:5 A:B with A 0.1 M ammonium acetate (diluted from molecular biology grade, 7.5 M, Merck Sigma-Aldrich, Hoeilaart, Belgium) in water (HiPerSolv CHROMANORM^®^; VWR, Leuven, Belgium) and B methanol (HiPerSolv CHROMANORM^®^; VWR, Leuven, Belgium), 99.9:0.1 A:B to 95:5 A:B to 72:28 A:B for 5 min, isocratic 72:28 A:B for 0.5 min, linear gradient 72:28 A:B to 0.1:99.9 A:B for 30 s, flow 0.4 µL min^−1^, column temperature 48 °C, injection volume 6 µL. For the aromatic O-glucosides, no pure standards are available. These derivatives were characterized and quantified by means of β-glucosidase treatment (5 U, from almonds, 2 U/mg, Merck Sigma-Aldrich, Hoeilaart, Belgium) on half of the samples following Werbrouck et al. [38]. Quantification was based on the differential results between before and after β-glucosidase treatment and the quantification of the corresponding aglycons.

Fraction B was acidified with 5 mL 6% formic acid and loaded on a pre-conditioned Bond Elut C18 solid phase extraction cartridge (Agilent Technologies, CA, USA). Pre-conditioning included ethanol (GPR RECTAPUR^®^; VWR, Leuven, Belgium), deionized water and 6% (*v*/*v*) formic acid (EMSURE^®^; Merck Sigma-Aldrich, Hoeilaart, Belgium) under 900 mbar (SC 920 G multi-user vacuum system; KNF, Verder, Aartselaar, Belgium). Compounds of interest were eluted with two times 3 mL of diethyl ether (AnalaR NORMAPUR^®^; VWR, Leuven, Belgium). The watery phase was removed and the eluent was dried under nitrogen (Zymark TurboVap LV Evaporator, Biotage, NC, USA, 37 °C). The sample was resolved in 100 µL acidified methanol (100 mL 100% MeOH + 5 drops HCl) and methylated by titration using freshly synthesized diazomethane at room temperature. The samples were dried under a nitrogen stream (Turbovap) and dissolved in 60 μL 10% (*v*/*v*) methanol, transferred to LC-MS vials and kept at 4 °C until analysis. IAA and IBA were analyzed as the corresponding methyl ester, using the internal tracer of IAA and taking into account the relative response using a calibration curve ranging from 10^−5^ to 10^−9^ M of both reference standard compounds (Olchemim, Olomouc, Czech Republic) dissolved in 10% (*v*/*v*) MeOH.

Fraction C was mixed with an equal amount of 12 N NaOH (EMSURE^®^; Merck Sigma-Aldrich, Hoeilaart, Belgium) for alkaline hydrolysis of all IAA- and IBA-conjugates (both amino acid conjugates as sugar conjugates). The sample was flushed with a water saturated nitrogen stream for 20 min and subsequently hydrolyzed for 180 min at 100 °C in a Pierce Reacti-Therm III (Thermo Fisher Scientific, Waltham, MA, USA). After hydrolysis, the sample was titrated with 2 M HCl (AnalaR^®^ NORMAPUR^®^; VWR, Leuven, Belgium) and diluted to a final volume of 10 mL with deionized water. Compounds of interest were loaded on a pre-conditioned Bond-Elut C18 SPE cartridge (Agilent Technologies, CA, USA) and diluted with two times 3 mL of diethyl ether. Further treatment and derivatization were undertaken as described above for fraction C. After this alkaline hydrolysis, IAA/IBA conjugates were analyzed as IAA-Me/IBA-Me in fraction C following the chromatographic conditions described for the analysis of IAA/IBA in fraction B.

All experiments were tested in a complete random block design, with 3 replications containing 12 shoots per replicate. Each block consisted of 3 replicates of each shoot position. Data were analyzed using the Kruskal–Wallis test, at *p* ≤ 0.05.

## 5. Conclusions

This study investigates and demonstrates one of the causes of heterogeneity in in vitro cultures of plants. When an explant is transferred to a new medium containing cytokinins, numerous detoxification mechanisms are activated, including reversible O-Glycosylation or irreversible N-Glycosylation. These findings indicate that the plant can deactivate mTR by shifting the hydroxy position to ortho or para.

As the discovery of CK metabolites progresses, more questions arise that require answers and explanations. Previous research has demonstrated that the proportions of N-glucosides vary across species [39]. This study further reveals that these ratios are influenced by the position of the explant. Additionally, by the distribution of N-glucosides, O-glucosides, and ribosides, that originate from the accumulation and the metabolism of the exogenously added mTR. The observed topophysical effect cannot be solely explained by analyzing endogenous ISCKs and auxin. However, the metabolism and distribution of the ARCKs offer an explanation.

Furthermore, this investigation showcased the potential of meta-topolin riboside in the micropropagation of *H. guayacan*, which could feasibly extend to other tropical woody plants. The study revealed that the plant produces various metabolites, each distributed and concentrated according to its original position. Further research will be published in the future to better understand the implications, such as a feeding experiment with labeled N^15^ mTR.

## Figures and Tables

**Table 1:** Morphological parameters of *H. guayacan* in vitro shoots developed from three different explant sections: (a) upper section; (b) middle section; (c) basal section.

	Number of Shoots	Length of Shoots (cm)	Number of Nodes/Shoot	Number of Nodes/Plant	Weight of Callus (g)	Length of Internodes (cm)
Upper section	1.00 ± 0.0 ^a^	2.64 ± 1.1 ^a^	5.35 ± 1.5 ^c^	5.35 ± 1.5 ^a^	0.38 ± 0.2 ^a^	0.51 ± 0.2 ^a^
Middle section	3.80 ± 1.3 ^c^	3.97 ± 1.1 ^b^	4.88 ± 1.5 ^b^	18.86 ± 6.5 ^c^	0.36 ± 0.2 ^a^	0.84 ± 0.2 ^c^
Basal section	2.53 ± 0.8 ^b^	2.67 ± 1.2 ^a^	3.94 ± 1.6 ^a^	10.15 ± 4.5 ^b^	0.51 ± 0.3 ^b^	0.72 ± 0.3 ^b^

Means followed by the same letter are not significantly different (*p* ≤ 0.05; Kruskal–Wallis test).

## Data Availability

Not applicable.
